# Key genes as stress indicators in the ubiquitous diatom *Skeletonema marinoi*

**DOI:** 10.1186/s12864-015-1574-5

**Published:** 2015-05-28

**Authors:** Chiara Lauritano, Ida Orefice, Gabriele Procaccini, Giovanna Romano, Adrianna Ianora

**Affiliations:** Stazione Zoologica Anton Dohrn, Villa Comunale, 80121 Napoli, Italy

**Keywords:** Diatoms, Stress-related genes, Molecular indicators, Nutrient starvation, Ocean acidification

## Abstract

**Background:**

The dense phytoplankton blooms that characterize productive regions and seasons in the oceans are dominated, from high to low latitudes and from coast line to open ocean, by comparatively few, often cosmopolitan species of diatoms. These key dominant species may undergo dramatic changes due to global climate change.

**Results:**

In order to identify molecular stress-indicators for the ubiquitous diatom species *Skeletonema marinoi*, we tested stress-related genes in different environmental conditions (i.e. nutrient starvation/depletion, CO_2_-enrichment and combined effects of these stressors) using RT-qPCR. The data show that these stressors impact algal growth rate, inducing early aging and profound changes in expression levels of the genes of interest.

**Conclusions:**

Most analyzed genes (e.g. antioxidant-related and aldehyde dehydrogenases) were strongly down-regulated which may indicate a strategy to avoid unnecessary over-investment in their respective proteins. By contrast, key genes were activated (e.g. HSPs, GOX) which may allow the diatom species to better cope with adverse conditions. We propose the use of this panel of genes as early bio-indicators of environmental stress factors in a changing ocean.

**Electronic supplementary material:**

The online version of this article (doi:10.1186/s12864-015-1574-5) contains supplementary material, which is available to authorized users.

## Background

Diatoms are eukaryotic unicellular plants that constitute one of the major components of marine phytoplankton [1] comprising up to 40% of annual productivity at sea [2] and representing up to 25% of global carbon-fixation [3]. Natural (e.g. cold/heat and nutrient limitation) and/or anthropogenic (e.g. ocean acidification and pollutants) factors may alter the physiology and survival of diatoms, thereby influencing current levels of ocean primary productivity [4,5]. Here we use Reverse Transcription-quantitative PCR (RT-qPCR) to identify key genes as indicators of defense processes activated in response to various stressful conditions in the ubiquitous diatom *Skeletonema marinoi,* a species which forms massive blooms in many of the world’s coastal oceans [6-8]. Previous studies on *S. marinoi* have focused on its genetic structure [8], secondary metabolite production [9], physiological response to nutrient limitation [10] and interactions with zooplankton species [6,7,11,12]. This is the first study focusing on stress-related genes as health-status indicators in this diatom species.

The stress conditions tested were senescence, silicic acid limitation/starvation, CO_2_-enrichment and the combination of these conditions. Si-concentrations vary extensively in the world’s oceans, depending on the regions and depths analyzed [13] and it is generally assumed that biogenic silicate content of some diatoms decreases under ocean acidification conditions [14]. Since diatoms incorporate inorganic silicon into the cell as silica [15] and use it to construct their outer cell wall, the availability and distribution of silicic acid can strongly modify diatom growth and population dynamics [16]. Hence we hypothesized that the combination of ocean acidification and variations in silicic acid availability would potentially negatively impact diatom growth and modify stress gene expression levels [14,16-18].

Current levels of atmospheric CO_2_ are predicted to be more than double by 2100 (Intergovernmental Panel on Climate Change IPCC 2007). Studies have reported contrasting results on the effects of ocean acidification in different phytoplankton species. Some studies speculate that larger diatoms will be favored [15], while others suggest that diatoms may age faster and undergo senescence/aging or even disappear in some areas of the oceans [18].

In order to test the effects of these two stressors (changes in silicic acid and CO_2_ levels), we selected a panel of genes involved in both first [19] and second lines of defense [20,21]: ATP-binding cassette transporter (ABC), aldehyde dehydrogenases (ALDH), succinate dehydrogenase (SSD), betaine ALDH (BALDH), glutathione synthase (GSH-S), glutathione peroxidase (GPX), glutathione reductase (GR), glutathione S-transferase (GST), catalase (CAT), superoxide dismutase (SOD), ascorbate peroxidase (AP) and tocopherol cyclase (TOCC) (see Additional file 1 for details). We also analyzed glycolate oxidase (GOX), an enzyme involved in stress resistance in the higher plant *Arabidopsis* thaliana [22] and some of the major families of heat shock proteins (HSP70, HSP90 and luminal binding protein or LBP), molecular chaperones known to be involved in the stress response induced by various environmental factors such as heat, cold, hypoxia, UV radiation and aging [23]. Activation of all these genes is known to help adjust cellular physiology and metabolism to changing conditions by offering protection against cell damage or death in other organisms [19-23].

## Results

### Aging

**Figure 1:**
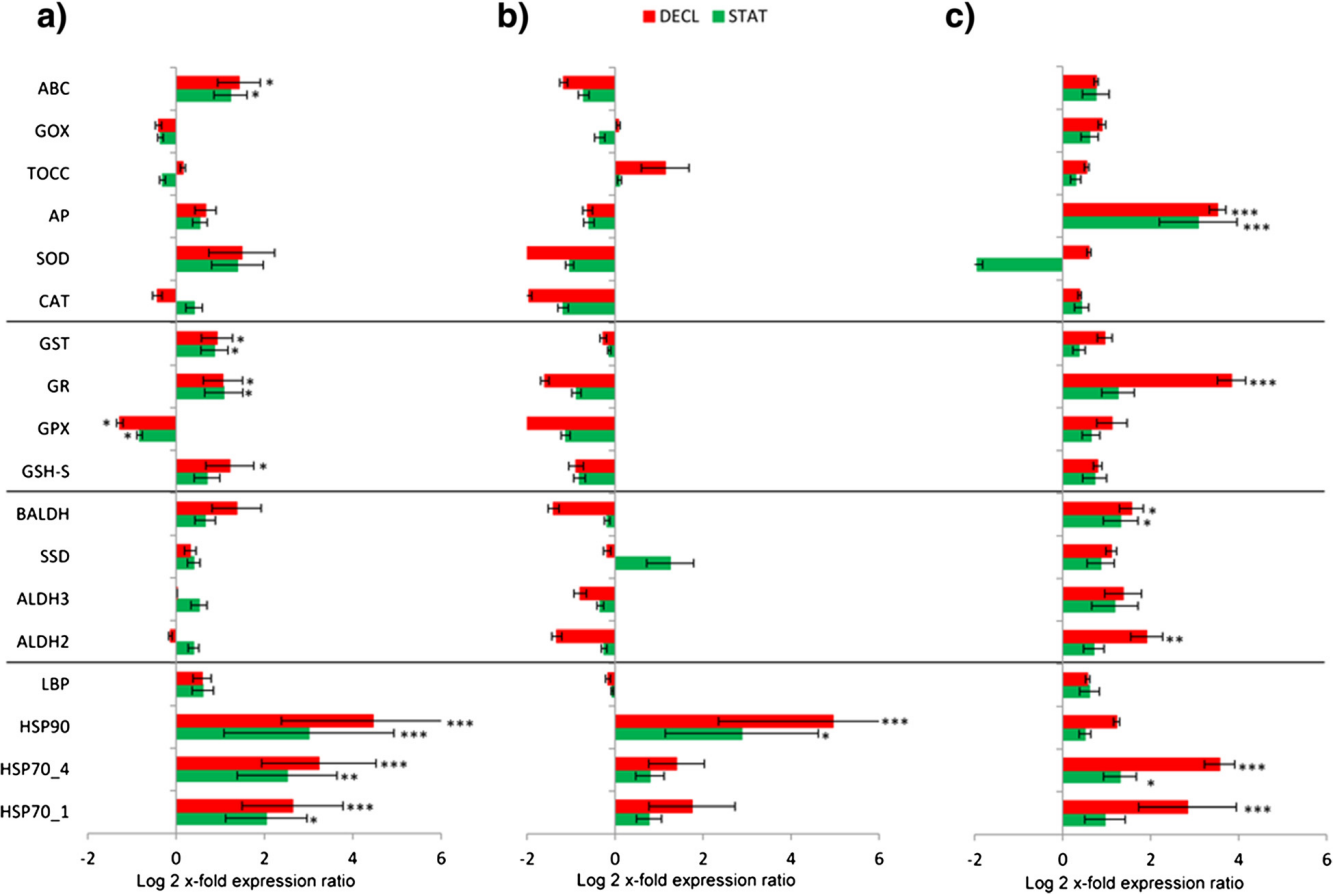
Aging gene expression in the diatom *Skeletonema marinoi.* Expression levels of heat shock proteins, aldehyde dehydrogenases, glutathione-related enzymes and other antioxidants in *S. marinoi* during stationary (STAT; green bars) and declining (DECL; red bars) growth phases in nutrient complete medium (normal condition, **a**), Si-starvation **(b)** and CO_2_-enriched/Si-starvation **(c)**, using their own exponential phase as control (x-axis; * for p < 0.05, ** for p < 0.01 and *** for p < 0.001). Data are represented as log2 x-fold expression ratio ± SD. Gene abbreviations used are: Heat shock proteins (HSP), luminal binding protein (LBP), aldehyde dehydrogenases (ALDH), succinate dehydrogenase (SSD), betaine ALDH (BALDH), glutathione synthase (GSH-S), glutathione peroxidase (GPX), glutathione reductase (GR), glutathione S-transferase (GST), catalase (CAT), superoxide dismutase (SOD), ascorbate peroxidase (AP), tocopherol cyclase (TOCC), glycolate oxidase (GOX), ATP-binding cassette transporter (ABC).

Here we show that all the stress conditions tested (nutrient starvation/depletion, CO_2_-enrichment and combinations) induced early aging in *S. marinoi*, as shown by net growth rates, which were 0.76 ± 0.05 d^-1^ for Si-starvation, 0.16 ± 0.01 d^-1^ for Si-depletion, 0.78 ± 0.005 d^-1^ for CO_2_-enrichment and 0.59 ± 0.007 d^-1^ for CO_2_-enrichment in combination with Si-starvation (referred to as CO_2_-Si) conditions, compared to the control 0.88 ± 0.02 d^-1^ (considering the growth rate between day 1 and day 7 for each condition). At the gene level, HSP expression levels increased in the stationary (STAT) and decline (DECL) phases of the growth curves in each studied condition (see Figure 1). Gene expression levels in the exponential phase (EXP) have been used as control condition for each RT-qPCR analysis shown in Figure 1 (the control condition is represented by the x-axis in the bar graph). The increase was time dependent, with the highest up-regulation in the declining phase of growth. In particular, in the growth curve of algae cultured with complete medium (Figure 1a), HSP70_1 and HSP70_4 were 2-fold up-regulated (p < 0.05 and p < 0.01, respectively), while HSP90 was significantly 3-fold up-regulated (p < 0.001) in STAT. In the DECL phase, HSP70_1 and HSP70_4 were 3-fold up-regulated (p < 0.001), while HSP90 expression levels increased by 4-fold (p < 0.001). The same pattern was observed for HSP expression level in the growth curve of algae grown in Si-starvation (Figure 1b) and in CO_2_-Si conditions (Figure 1c). Also in these cases, there was a time-dependent increase, but the up-regulation was statistically significant only for HSP90 (p < 0.05 in STAT and p < 0.001 in DECL) in Si-starvation (Figure 1b) and for HSP70_1 (p < 0.001 in DECL) and HSP70_4 (p < 0.05 in STAT and p < 0.001 in DECL) in CO_2_-Si condition (Figure 1c). Aging did not induce variations in other gene categories, with some exceptions. The Glutathione-related enzymes GSH-S, GR and GST expression levels were higher in STAT and DECL phases in algae grown in complete medium (p > 0.05, see Figure 1a for details), while GPX was down-regulated in both phases (p < 0.01) compared to the EXP one (Figure 1a). GR also was significantly up-regulated in DECL of the CO_2_-Si growth curve (p < 0.001; Figure 1c). ABC also increased in STAT and DECL in the complete medium curve (p < 0.05; Figure 1a). AP and BALDH increased in STAT and DECL (p < 0.001 for AP in both STAT and DECL, and p < 0.01 for BALDH in both STAT and DECL), GR and ALDH2 in DECL (p < 0.001 and p < 0.01, respectively) in the CO_2_-Si growth curve (Figure 1c).

### Nutrient starvation/depletion

**Figure 2:**
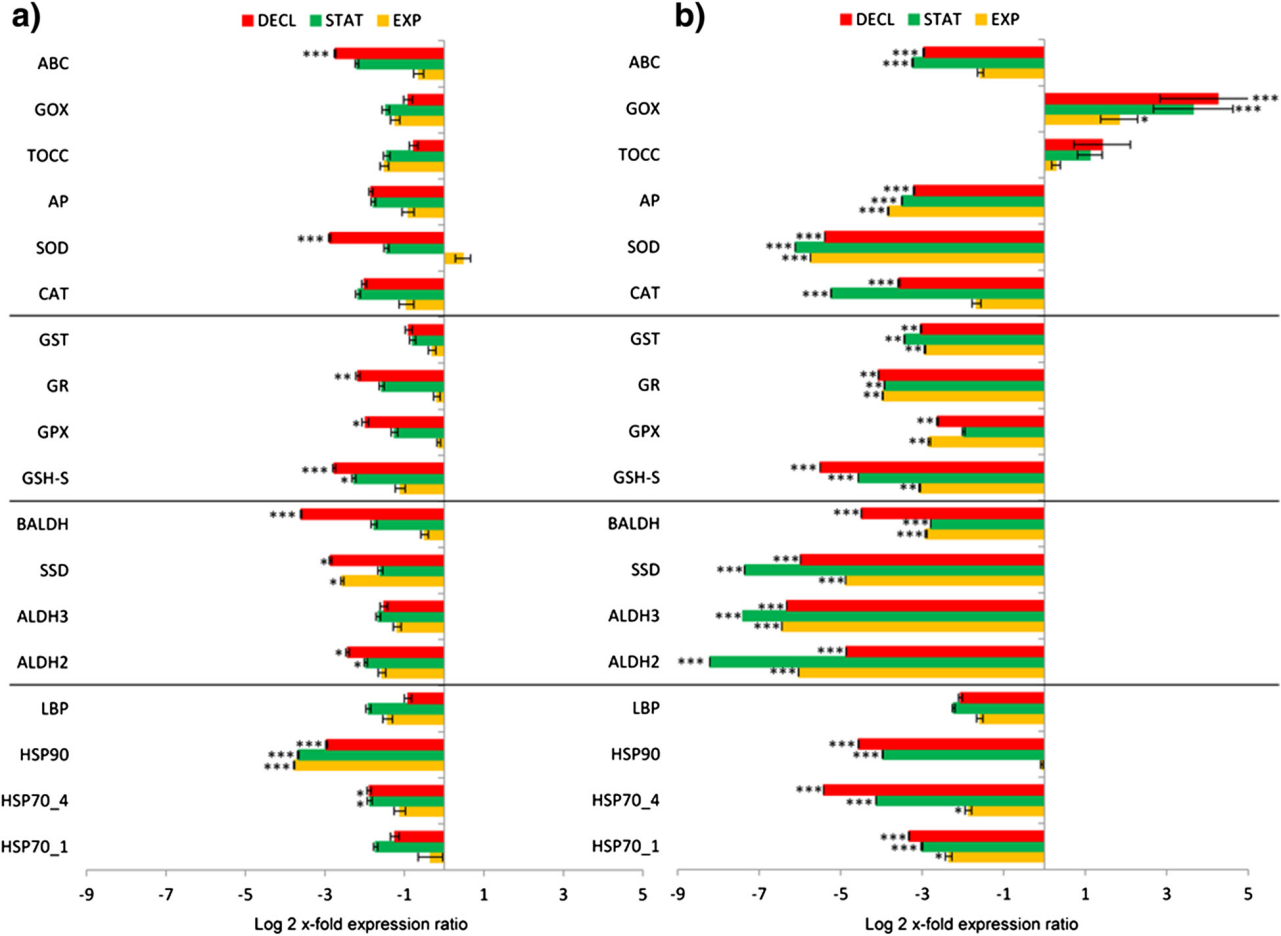
Gene expression in *Skeletonema marinoi* grown in Si-starvation or depletion conditions. Expression levels of heat shock proteins, aldehyde dehydrogenases, glutathione-related enzymes and other antioxidants in *S. marinoi* during exponential, stationary and declining growth phases (EXP, STAT, DECL) in **(a)** Si-starved or **(b)** Si-depleted media, using *S. marinoi* grown in complete medium as control (x-axis; * for p < 0.05, ** for p < 0.01 and *** for p < 0.001). Data are represented as log2 x-fold expression ratio ± SD. Gene abbreviations used are: Heat shock proteins (HSP), luminal binding protein (LBP), aldehyde dehydrogenases (ALDH), succinate dehydrogenase (SSD), betaine ALDH (BALDH), glutathione synthase (GSH-S), glutathione peroxidase (GPX), glutathione reductase (GR), glutathione S-transferase (GST), catalase (CAT), superoxide dismutase (SOD), ascorbate peroxidase (AP), tocopherol cyclase (TOCC), glycolate oxidase (GOX), ATP-binding cassette transporter (ABC).

Figure 2a shows gene expression changes in *S. marinoi* grown in Si-starvation, while 2b in Si-depletion condition. For both RT-qPCR analyses, gene expression levels of algae grown in complete medium have been used as control condition (in the bar graph the control condition is represented by the x-axis). In Si-starvation, there was the down-regulation of almost all the genes of interest (Figure 2a). HSP90 and SSD were the only genes that were down-expressed in EXP (p < 0.001 and p < 0.05, respectively). HSP70_4, HSP90, ALDH2 and GSH-S were down-regulated in STAT (p < 0.001 for HSP90 and p < 0.05 for the others). In DECL, many genes showed decreased expression levels: HSP70_4, HSP90,, ALDH2, SSD, BALDH, GSH-S, GPX, GR, SOD and ABC (p < 0.05 for HSP70_4, ALDH2, SSD and GPX, p < 0.01 for GR and p < 0.001 for the others). HSP90 was the most affected gene, with a decrease of more than 4-fold. In Si-depletion condition, the down-regulation was even more pronounced, with a reduction of more than 8-fold for ALDH2 (Figure 2b). HSPs, aldehyde dehydrogenases and antioxidants were impaired. GOX was the only exception and was significantly up-regulated in EXP, STAT and DECL (p < 0.05 for EXP and p < 0.001 for STAT and DECL). The increase was a time-dependent with maximum up-regulation of more than 4-fold in DECL (Figure 2b).

### CO_2_-enrichment and combinations

**Figure 3:**
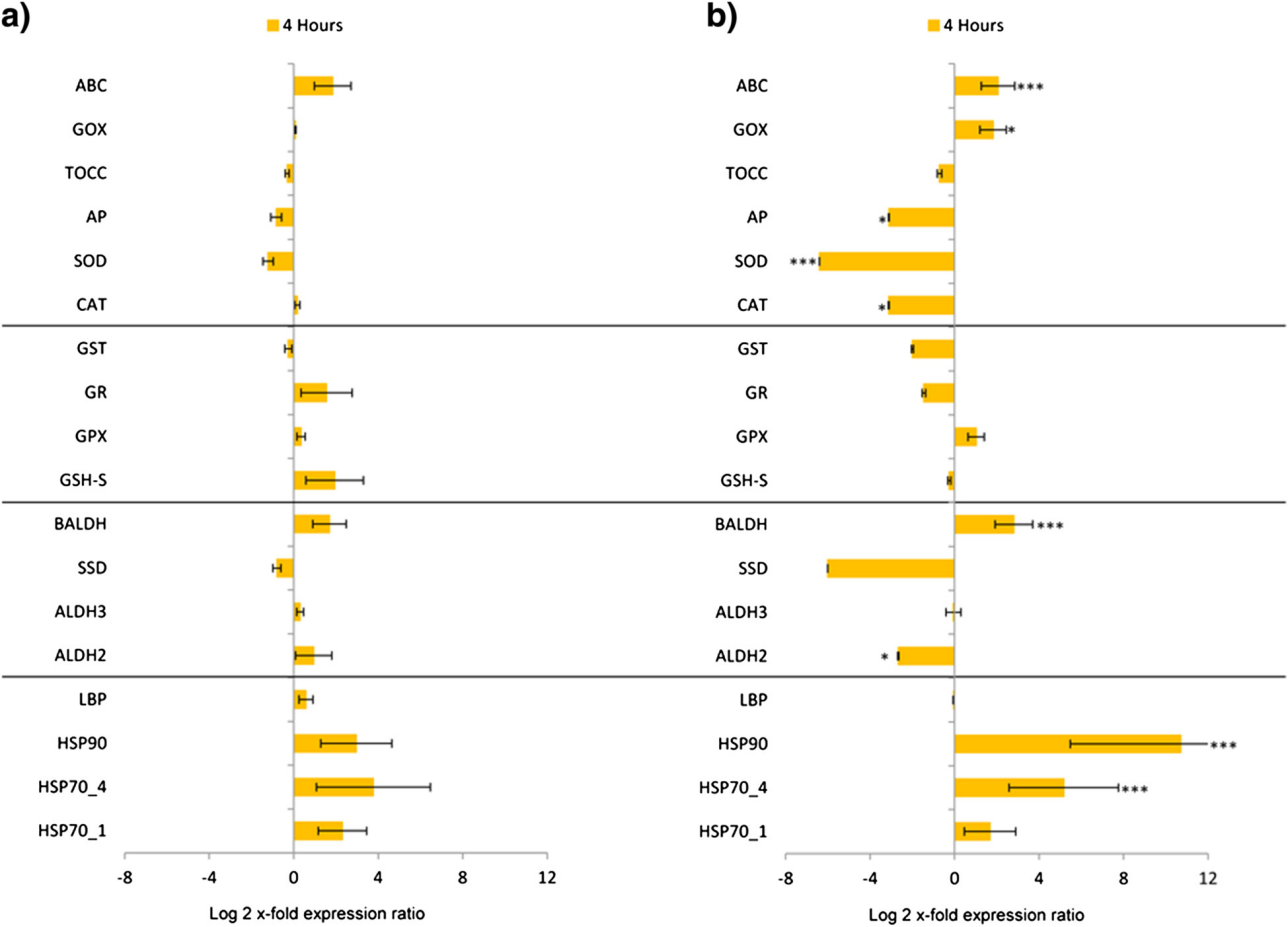
Gene expression in *Skeletonema marinoi* grown four hours in CO_2_-enriched or CO_2_-enriched Si-starved medium. Expression levels of heat shock proteins, aldehyde dehydrogenases, glutathione-related enzymes and other antioxidants in *S. marinoi* grown four hours in CO_2_-enriched medium **(a)** or in CO_2_-enriched/Si-starvation medium **(b)**. *S. marinoi* grown in complete medium was used as control (x-axis; * for p < 0.05, ** for p < 0.01 and *** for p < 0.001). Data are represented as log2 x-fold expression ratio ± SD. Gene abbreviations used are: Heat shock proteins (HSP), luminal binding protein (LBP), aldehyde dehydrogenases (ALDH), succinate dehydrogenase (SSD), betaine ALDH (BALDH), glutathione synthase (GSH-S), glutathione peroxidase (GPX), glutathione reductase (GR), glutathione S-transferase (GST), catalase (CAT), superoxide dismutase (SOD), ascorbate peroxidase (AP), tocopherol cyclase (TOCC), glycolate oxidase (GOX), ATP-binding cassette transporter (ABC).

**Figure 4:**
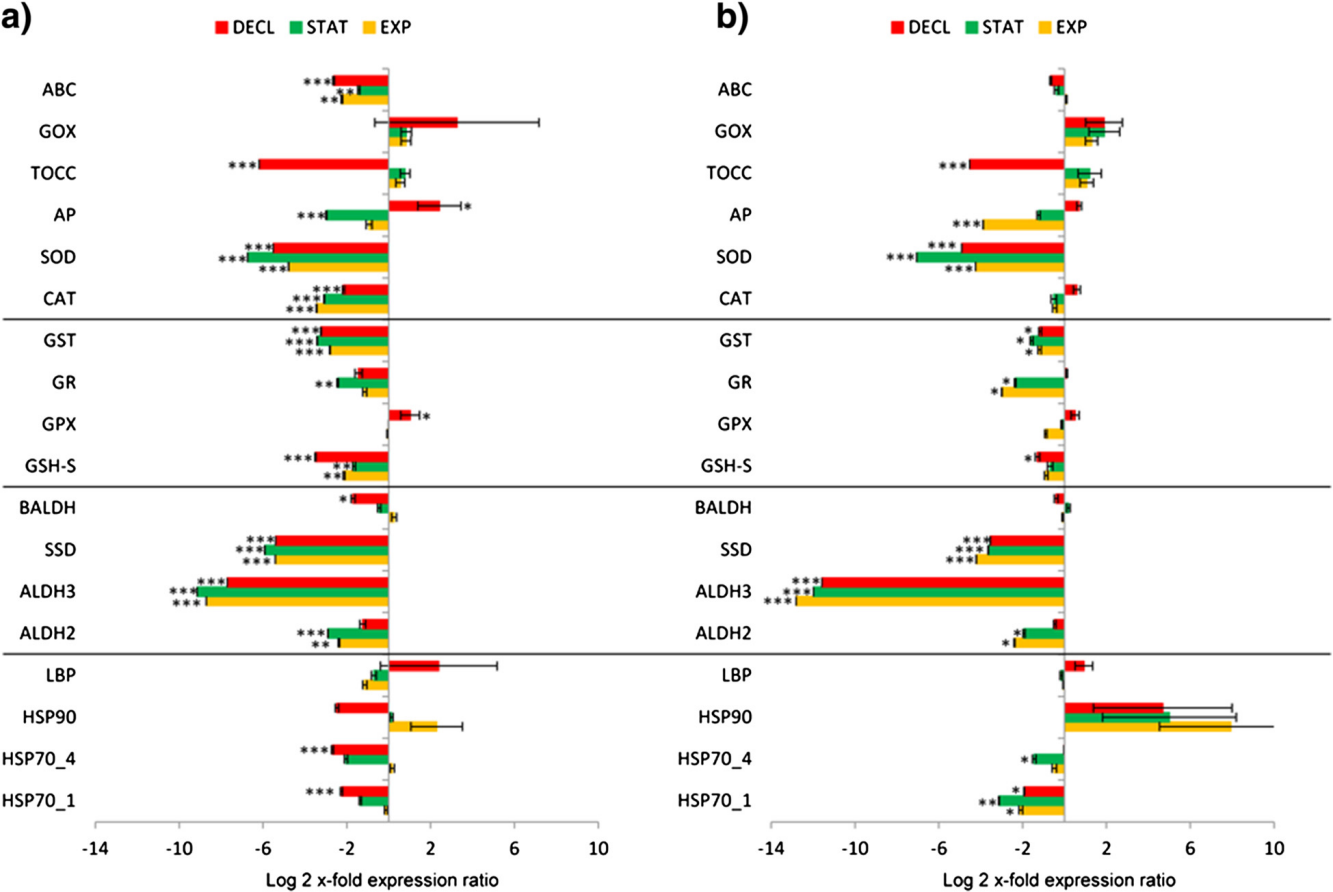
Gene expression in *Skeletonema marinoi* grown in CO_2_-enriched or CO_2_-enriched Si-starved medium. Expression levels of heat shock proteins, aldehyde dehydrogenases, glutathione-related enzymes and other antioxidants in *S. marinoi* during exponential, stationary and decline growth phases (EXP, STAT, DECL) in CO_2_-enriched medium **(a)** or in CO_2_-enriched/Si-starvation medium **(b)**, using *S. marinoi* grown in complete medium as control (x-axis; * for p < 0.05, ** for p < 0.01 and *** for p < 0.001). Data are represented as log2 x-fold expression ratio ± SD. Gene abbreviations used are: Heat shock proteins (HSP), luminal binding protein (LBP), aldehyde dehydrogenases (ALDH), succinate dehydrogenase (SSD), betaine ALDH (BALDH), glutathione synthase (GSH-S), glutathione peroxidase (GPX), glutathione reductase (GR), glutathione S-transferase (GST), catalase (CAT), superoxide dismutase (SOD), ascorbate peroxidase (AP), tocopherol cyclase (TOCC), glycolate oxidase (GOX), ATP-binding cassette transporter (ABC).

**Figure 5:**
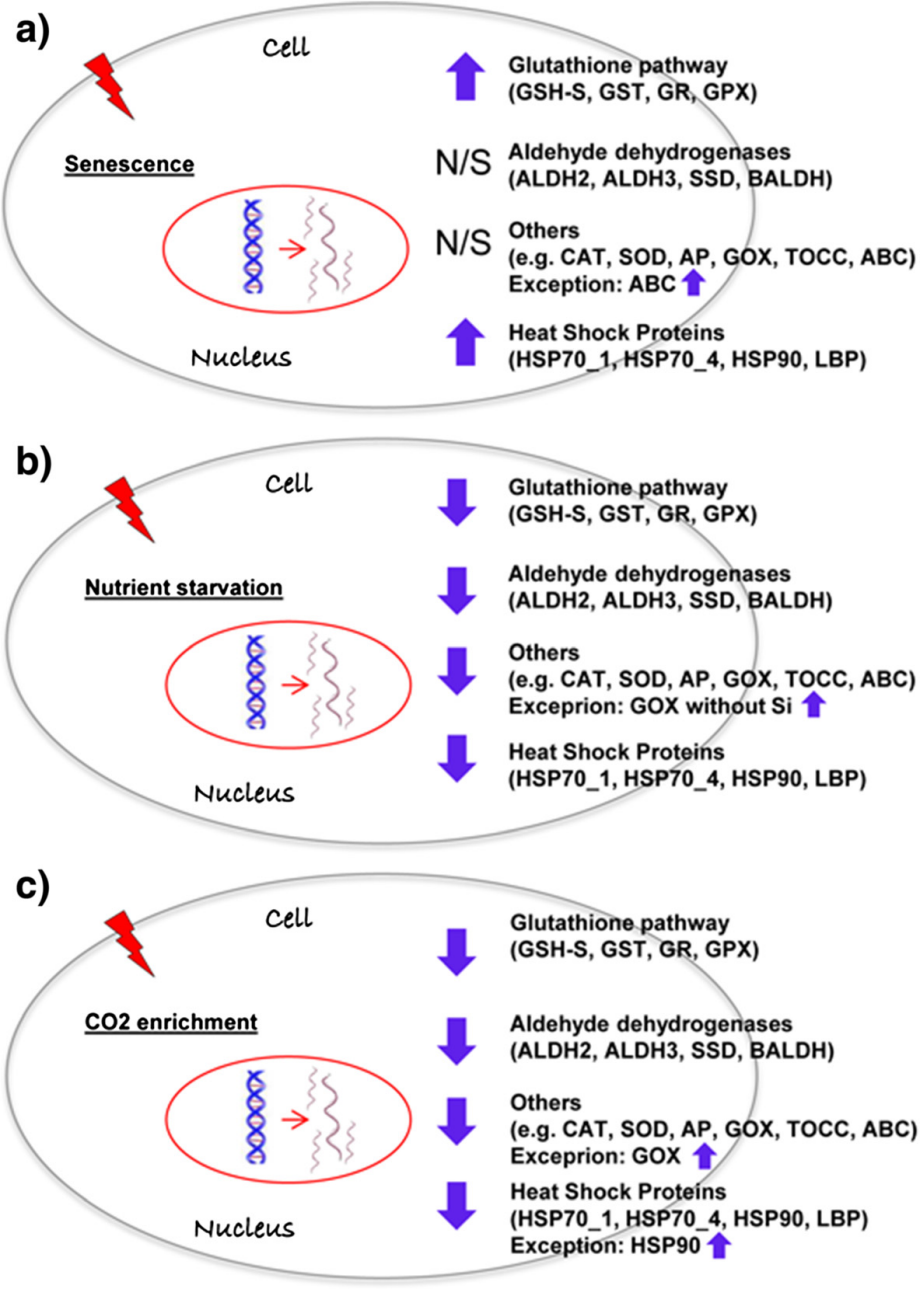
Gene expression overview of *S. marinoi* with aging, nutrient starvation and CO_2_ enrichment. Summary of expression level results of the studied gene categories (Heat shock proteins, Aldehyde dehydrogenases, Glutathione-related enzymes, and other antioxidants) in different tested conditions: Aging **(a)**, Nutrient starvation (Silicic acid starvation/depletion, **b**), CO_2_ bubbling (for 4 h or continuously, **c**). Arrows indicate up- or down-regulation of selected genes. N/S indicates that non-significant gene expression changes were observed. Gene abbreviations used are: Heat shock proteins (HSP), luminal binding protein (LBP), aldehyde dehydrogenases (ALDH), succinate dehydrogenase (SSD), betaine ALDH (BALDH), glutathione synthase (GSH-S), glutathione peroxidase (GPX), glutathione reductase (GR), glutathione S-transferase (GST), catalase (CAT), superoxide dismutase (SOD), ascorbate peroxidase (AP), tocopherol cyclase (TOCC), glycolate oxidase (GOX), ATP-binding cassette transporter (ABC).

CO_2_-bubbling for only four hours, did not induce different expression level changes in the genes of interest (Figure 3a), but algae grown in CO_2_-Si condition for four hours showed increased levels of HSP70_4, HSP90, BALDH, GOX and ABC (p < 0.05 for GOX and p < 0.001 for the others) and a decrease in ALDH2, CAT, SOD and AP (p < 0.001 for SOD and p < 0.05 for the others) (Figure 3b). Finally, using CO_2_-enriched medium for the entire growth curve (Figure 4a) there was the down-regulation of almost all the genes, with the lowest reduction in ALDH3 (10-x fold). HSP70_1 and HSP70_4 were down-regulated in DECL (p < 0.001), ALDH2 in EXP and STAT (p < 0.01 and 0.001, repectively), ALDH3 and SSD in EXP, STAT and DECL (p < 0.001 for all) and BALDH only in DECL (P < 0.05). For the glutathione-related genes and others, GSH-S, GST, CAT, SOD and ABC decreased in STAT (p < 0.01 for GSH-S and ABC, p < 0.001 for the others). GSH-S, GR, CAT, SOD, AP and ABC decreased in STAT (p < 0.001 for GST, SOD and AP, p < 0.01 for the others), whereas GSH-S, GST, CAT, SOD, TOCC and ABC decreased in DECL (p < 0.001 for all). Only AP and GPX showed increased expression levels (p < 0.05 for both). When this experimental condition was combined with Si-starvation (Figure 4b) only down-regulation patterns were observed, with the lowest reduction of more than 13-fold (ALDH3; Figure 4b). In EXP, HSP70_1, ALDH2, ALDH3, SSD, GR, GST, SOD and AP were reduced (p < 0.001 for ALDH3, SSD, SOD and AP, p < 0.05 for the others), in STAT, HSP70_1, HSP70_4, ALDH2, ALDH3, SSD, GR, GST and SOD (p < 0.05 for HSP70_4, ALDH2, GR and GST, p < 0.01 for HSP70_1 and p < 0.001 for the others) were reduced while in DECL, HSP70_1, ALDH3, SSD, GSH-S, GST, SOD and TOCC (p < 0.5 for HSP70_1, GSH-S, GST, p < 0.001 for the others) were reduced. For some genes, changes were not significant (see asterisks in Figures). All the results are summarized in Figure 5.

## Discussion

Stress is associated with the generation of reactive oxygen species (ROS). In low quantities, ROS are rapidly converted to less reactive forms, but when present in abnormally high quantities, they can be very damaging to DNA, RNA and proteins. Several studies have reported an increase in the activity of antioxidant ROS-scavenging enzymes also with aging [24], but this has not yet been shown in marine diatoms. Our results indicate that some of the antioxidants (especially glutathione-related enzymes) were up-regulated during aging (in both STAT and DECL) in *S. marinoi* in normal (Figure 1a) and CO_2_-Si conditions (Figure 1c). In addition, our results show that HSP expression levels increase with aging in all conditions tested. An increase in HSPs with aging was first observed in *Drosophila* melanogaster [25] due to an increase in misfolded proteins and the expression of chaperonines (e.g. HSPs) for protein re-folding. HSP expression levels increased in both STAT and DECL phases of *S. marinoi* growth (compared to their respective EXP phases, represented in Figure 1 by x-axis) in normal (Figure 1a), Si-starvation (Figure 1b), CO_2_-Si (Figure 1c) and CO_2_ enrichment (see Additional file 2) conditions. The increase was time-dependent, with the highest up-regulation in the declining phase (Figure 1, in red). On the contrary, aging did not induce changes in expression levels in the other gene categories studied, e.g. ALDHs and other antioxidants. These results suggest the potential use of HSPs and some antioxidants (especially the glutathione-related enzyme GR) as indicators of aging in marine diatoms.

Stress caused by silicic acid and iron starvation induced early aging in the diatom *Thalassiosira* pseudonana [26]. Here we show that nutrient starvation/depletion (silicic acid) and CO_2_-enrichment induced early aging in *S. marinoi*, as shown by net growth rates. These data suggest that the strongest stressful condition for *S. marinoi* survival in the present study was the absence of silicates.

We then analysed gene expression levels under two nutrient conditions: reduction of silicic acid in the culturing medium (from 107 μM of the control to 36 μM) and complete Si-removal (Figure 2a and b, respectively). Nutrient depletion/reduction did not induce activation of HSPs and the majority of tested genes. In both experiments, expression levels of HSPs, ALDHs, GSH-S, GR, GPX and GST were significantly reduced without silicic acid or in Si-starvation conditions compared to controls (Figure 2a and b). Interestingly, a decrease in expression levels was time-dependent in Si-starvation conditions only for GSH-S, GR, GPX and GST, with highest expression in the exponential phase (EXP) and lowest in the declining phase (DECL) (Figure 2a). Gene down-regulation in cells grown without silicic acid was even more pronounced. In order to understand if the observed down-regulation is Si-limitation specific or is common for other nutrient limitation conditions, we also cultivated *S. marinoi* in phosphate-starvation (P-starvation). Results showed that also in this case there was a significant down-regulation of the genes of interest, except for the up-regulation of GOX, of the antioxidant GPX and the chaperonine HSP90 (see Additional file 3). Overall, this reduction in gene expression in response to nutrient starvation may indicate a cell strategy to avoid unnecessary over-investment in the respective proteins, similar to the depression in protein synthesis that occurs in other organisms during environmental stress, considered a substantial bioenergetic saving process [27].

CO_2_-enrichment is reported to induce contrasting results in phytoplankton, with more or less tolerant species, such as *Cyanidium caldarium* and *Tetraselmis sp*, respectively [28]. Engel *et al*. [29] carried out a mesocosm experiment to document the effect of CO_2_ concentration on a bloom of the haptophyte algae *Emiliania huxleyi*, which supported earlier findings of reduced calcification at higher CO_2_ concentrations. Hutchins *et al*. [30] studied dinitrogen (N_2_) and CO_2_ fixation rates under future CO_2_ scenarios in the marine cyanobacteria *Trichodesmium*, which contributes a large fraction of the new nitrogen entering the oligotrophic oceans. They suggested that predicted elevated CO_2_ levels in the future could alter current marine N and C cycles. Here, we tested the effect of CO_2_-enrichment, alone or in combination with Si-starvation, after four hours and after prolonged exposure (7 days). After 4 hours of CO_2_-enrichment, various genes were up-regulated (e.g. HSPs, ALDHs and some antioxidants; Figure 3a), but after prolonged exposure (Figure 4a) these levels were significantly reduced, suggesting that the activation of these genes may be only a short-term stress signal. Copper stress induced a similar response in the diatom *Thalassiosira* pseudonana [31]. This diatom exhibited a rapid induction of certain Cu-related genes to elevated concentrations of copper (after 1 h-exposure), but this response was attenuated over 24 h of continuous exposure. The down-regulation observed in CO_2_-enrichment experiments (Figures 3b, 4a and 4b) may be an energetic cost-saving strategy used by organisms to allow restoration of homeostasis and survival [27].

In contrast to most of the stress-related genes studied here, glycolate oxidase (GOX) was up-regulated in Si-depletion, after prolonged CO_2_-bubbling, CO_2_-Si, and P-starvation (Figures 2b, 4a, 4b). Glycolate oxidizing enzyme is known to play a key role in photorespiratory carbon metabolism responsible for reducing carbon loss and oxidative damage [32]. However, photorespiration also plays other roles in higher plants e.g. amino acid metabolism, nitrate reduction, stress resistance, and signal transduction [32,33]. Our results suggest a specific protective antioxidant activity for GOX that does not always imply a change in C metabolism, as also found in the higher plants *Arabidopsis thaliana* and *Oryza* sativa [32,33].

## Conclusions

Our results provide first insights on early warning signals for environmental stressful conditions, such as those predicted under a Global Climate Change scenario, in the ubiquitous diatom *Skeletonema marinoi* (as summarized in Figure 5). Nutrient starvation/depletion, CO_2_-enrichment and the combined effect of stressors, impact the growth rate of this species, inducing early aging. HSPs behave as signals of senescence/aging (Figure 5a) and short-term exposure to stress (i.e. HSP90; Figure 5c) whereas the antioxidant GOX as a signal of strong nutrient depletion (silicic acid) and/or exposure to high CO_2_ concentration with or without Si-starvation (Figure 5b,c).

## Methods

### Cell culturing and harvesting

*Skeletonema marinoi* (CCMP2501) was grown in Guillard’s f/2 medium [34] in two-liter polycarbonate bottles (each experiment was performed in triplicate) constantly bubbled with air filtered through 0.2 μm membrane filters. For the Si- and P-starvation experiments medium was prepared with low concentration of silicic acid (36 μM Si(OH)_4_) and with low concentration of phosphate (0.5 μM PO_4_^2-^), respectively. CO_2_-enrichment (alone or in combination with Si-starvation, referred to as CO_2_-Si) was obtained through continuous CO_2_-bubbling for few hours (4 h) or during the entire growth curve (until day 7), using the CO_2_ dispenser RuWal (RuWal aquatech). The experimental pH value was 6.5 (control pH was about 8). Cultures were kept in a climate chamber at 19°C on a 12:12 h light:dark cycle at 100 μmol photons m^-2^ s^-1^. Initial cell concentrations were about 5000 cells/mL for each experiment and culture growth rate was monitored, using the equation for net growth estimates [35]. A 50 mL culture aliquot was sampled each day (from day 3 to day 7 after inoculation, at the same time of the day to avoid possible interference by intrinsic circadian rhythms), and centrifuged for 30 minutes at 4°C at 3900 g (Eppendorf, 5810R). The pellet was re-suspended in 500-800 μL of TRIZOL© (Invitrogen, Carlsbad, CA), incubated for 2-3 minutes at 60°C until completely dissolved and kept at -80°C until RNA extraction.

### RNA extraction and cDNA synthesis

For RNA extraction, cells were lysed using half a spatula of glass beads (about 200 mg; Sigma-Aldrich, Milan, Italy) for each 2 mL tube, incubating and mixing tubes for 10 min at 60°C and maximum speed in the Thermo Shaker BS100 (Biosan). RNA was then extracted using TRIZOL© manufacturer’s protocol. RNA quantity and purity were assessed by Nano-Drop (ND-1000 UV-Vis spectrophotometer; NanoDrop Technologies) monitoring the absorbance at 260 nm and the 260/280 nm and 260/230 nm ratios (Both ratios were about 2.0). RNA quality was evaluated by gel electrophoresis that showed intact RNA, with sharp ribosomal bands. 500 ng of each RNA were retrotranscribed into complementary DNA (cDNA) with the iScriptTM cDNA Synthesis Kit (BIORAD, Hercules, CA) following the manufacturer’s instructions.

### Oligo design

Primers for genes of interest (GOI) were designed using the software Primer3 v. 0.4.0 (http://frodo.wi.mit.edu/primer3/) considering sequences from the transcriptome of the diatom *Skeletonema marinoi* deposited in the public database CAMERA (http://camera.crbs.ucsd.edu/mmetsp/list.php) and in iMicrobe, interactive query tool for microbial data (http://data.imicrobe.us/sample/view/1867). See Additional file 1 for selected GOI, their functions, primers’ sequences, efficiencies and correlation factor. Primers were optimized in a GeneAmp PCR System 9700 (Perkin Elmer). For a detailed description see [11].

### Reverse transcription-quantitative polymerase chain reaction (RT-qPCR)

In order to normalize expression levels of specific GOI, a panel of putative reference genes (RGs), was first screened in the experimental conditions: entire growth curve in normal condition (data not shown), total Si-experiment (both growth without silicic acid and silica starvation), total CO_2_ experiment (growth in CO_2_-enriched water for a few hours or along the entire growth curve), CO_2_-Si and P-starvation. Three different algorithms were utilized to identify the best RGs in our experimental design: BestKeeper [36], geNorm [37] and NormFinder [38]. See Additional file 4 for specific RGs for each experiment.

Serial dilutions of cDNA were used to determine GOI primer reaction efficiency (E) and correlation factor (R^2^) (see Additional file 1). Standard curves were generated with five dilution points by using the cycle threshold (Ct) value versus the logarithm of each dilution factor and using the equation E = 10^-1/slope^. RT-qPCR was performed in MicroAmp Optical 384-Well reaction plate (Applied Biosystem, Foster City, CA) with optical adhesive covers (Applied Biosystem) in a Viia7 real-time PCR system (Applied Biosystem) and using the fluorescent dye Fast Start SYBR Green Master Mix (Roche, Indianapolis, IN). The PCR volume for each sample was 10 μl, with 5 μl of Fast Start SYBR Green Master Mix, 1 μl of cDNA template (1:50 template dilution) and 0.7 pmol/mL for each oligo. The RT-qPCR thermal profile was obtained using the following procedure: 95°C for 20 s, 40 times 95°C for 1 s, and 60°C for 20 s. The program was set to reveal the melting curve of each amplicon from 60 to 95°C, and read every 0.5°C. Only a single peak was identified in the melting-curve analyses of all genes, confirming a gene-specific amplification and the absence of primer-dimers. All RT-qPCR reactions were carried out in triplicate to capture intra-assay variability and included three no-template negative controls (NTC) for each primer pair. To study expression levels for each GOI relative to the most stable RGs, we used the REST tool (Relative Expression Software Tool) [39]. Control conditions changed depending on the specific experiment (See Figure legends for each specific case). Statistical analysis was performed using the Pair Wise Fixed Reallocation Randomisation test by REST [39] and GraphPad Prim statistic software, V4.00 (GraphPad Software).

### Availability of supporting data

Sequences of the transcriptome of the diatom *Skeletonema marinoi* are deposited in the public database CAMERA (http://camera.crbs.ucsd.edu/mmetsp/list.php) and in iMicrobe, interactive query tool for microbial data (http://data.imicrobe.us/sample/view/1867).

## Additional files

Additional file 1:
**Lists selected genes of interest, their abbreviations and functions, sequences of forward and reverse primers, efficiencies (E) and correlation factor (R).** Gene abbreviations used are: Heat shock proteins (HSP), luminal binding protein (LBP), aldehyde dehydrogenases (ALDH), succinate dehydrogenase (SSD), betaine ALDH (BALDH), glutathione synthase (GSH-S), glutathione peroxidase (GPX), glutathione reductase (GR), glutathione S-transferase (GST), catalase (CAT), superoxide dismutase (SOD), ascorbate peroxidase (AP), tocopherol cyclase (TOCC), glycolate oxidase (GOX), ATP-binding cassette transporter (ABC). Additional file 2:
**Aging gene expression in the diatom** ***Skeletonema marinoi*** **in the CO**_**2**_ **-enriched condition.** Expression levels of heat shock proteins, aldehyde dehydrogenases, glutathione-related enzymes and other antioxidants in *S. marinoi* during stationary (STAT; green bars) and declining (DECL; red bars) growth phases in the CO_2_-enriched condition, using the exponential phase as control (x-axis; * for p < 0.05, ** for p < 0.01 and *** for p < 0.001). Data are represented as log2 x-fold expression ratio ± SD. Gene abbreviations used are: Heat shock proteins (HSP), luminal binding protein (LBP), aldehyde dehydrogenases (ALDH), succinate dehydrogenase (SSD), betaine ALDH (BALDH), glutathione synthase (GSH-S), glutathione peroxidase (GPX), glutathione reductase (GR), glutathione S-transferase (GST), catalase (CAT), superoxide dismutase (SOD), ascorbate peroxidase (AP), tocopherol cyclase (TOCC), glycolate oxidase (GOX), ATP-binding cassette transporter (ABC). Additional file 3:
**Gene expression in** ***Skeletonema marinoi*** **grown in P-starved medium.** Expression levels of heat shock proteins, aldehyde dehydrogenases, glutathione-related enzymes and other antioxidants in *S. marinoi* during exponential, stationary and declining growth phases (EXP, STAT, DECL) in P-starved medium, using *S. marinoi* grown in complete medium as control (x-axis; * for p < 0.05, ** for p < 0.01 and *** for p < 0.001). Data are represented as log2 x-fold expression ratio ± SD. Gene abbreviations used are: Heat shock proteins (HSP), luminal binding protein (LBP), aldehyde dehydrogenases (ALDH), succinate dehydrogenase (SSD), betaine ALDH (BALDH), glutathione synthase (GSH-S), glutathione peroxidase (GPX), glutathione reductase (GR), glutathione S-transferase (GST), catalase (CAT), superoxide dismutase (SOD), ascorbate peroxidase (AP), tocopherol cyclase (TOCC), glycolate oxidase (GOX), ATP-binding cassette transporter (ABC). Additional file 4:
**Best reference genes in the experimental conditions analyzed.** Best reference genes as given by BestKeeper, NormFinder and Genorm analyses, for each of the experimental conditions studied: Silicic acid-starvation/depletion experiments, CO_2_ experiments (CO_2_ for four hours or for the entire growth curve), Si-starvation combined with CO_2_-enrichment and phosphate-starvation.

## Abbreviations

(ALDH): Aldehyde dehydrogenases
(AP): Ascorbate peroxidase
(ABC): ATP-binding cassette transporter
(BALDH): Betaine ALDH
(CO_2_): Carbon Dioxide
(CAT): Catalase
(CO_2_-Si): CO_2_-enrichment in combination with Si-starvation condition
(cDNA): Complementary DNA
(Ct): Cycle threshold
(DECL): Decline
(N_2_): Dinitrogen
(EXP): Exponential phase
(GOI): Genes of interest
(GPX): Glutathione peroxidase
(GR): Glutathione reductase
(GST): Glutathione S-transferase
(GSH-S): Glutathione synthase
(GOX): Glycolate oxidase
(HSP): Heat shock proteins
(LBP): Luminal binding protein
(NTC): No-template negative controls
(P-starvation): Phosphate starvation
(ROS): Reactive oxygen species
(RGs): Reference genes
PCR (RT-qPCR): Reverse Transcription-quantitative
(Si-starvation): Silicic acid starvation
(STAT): Stationary
(SSD): Succinate dehydrogenase
(SOD): Superoxide dismutase
(TOCC): Tocopherol cyclase
