# Supplementary figures and images for: Key genes as stress indicators in the ubiquitous diatom Skeletonema marinoi
Source: BMC Genomics. 2015 May 28;16(1):411. doi: 10.1186/s12864-015-1574-5 (PMC4445783; doi:10.1186/s12864-015-1574-5)

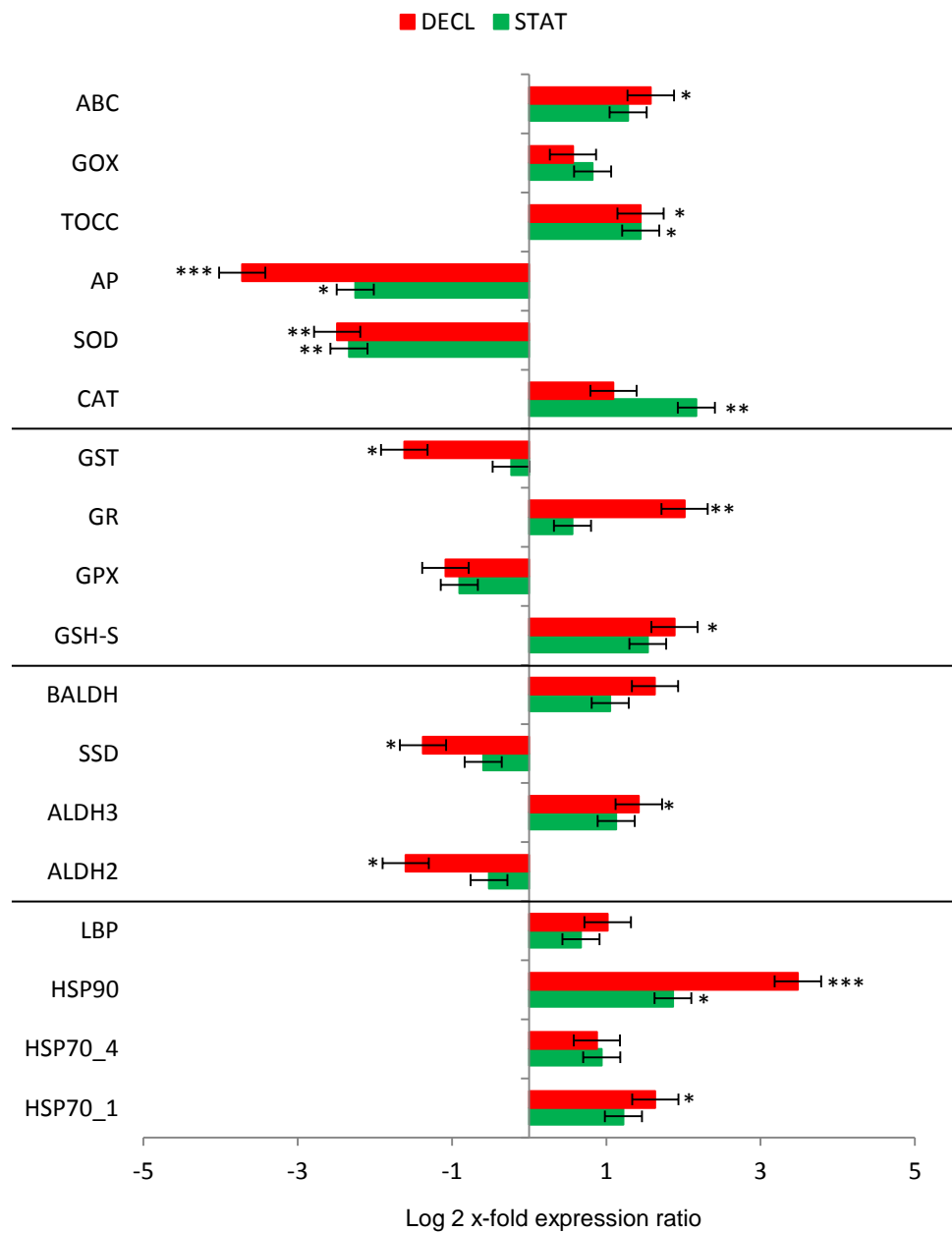

Supplement: Additional file 2: — Aging gene expression in the diatom Skeletonema marinoi in the CO 2 -enriched condition. Expression levels of heat shock proteins, aldehyde dehydrogenases, glutathione-related enzymes and other antioxidants in S. marinoi during stationary (STAT; green bars) and declining (DECL; red bars) growth phases in the CO2-enriched condition, using the exponential phase as control (x-axis; * for p < 0.05, ** for p < 0.01 and *** for p < 0.001). Data are represented as log2 x-fold expression ratio ± SD. Gene abbreviations used are: Heat shock proteins (HSP), luminal binding protein (LBP), aldehyde dehydrogenases (ALDH), succinate dehydrogenase (SSD), betaine ALDH (BALDH), glutathione synthase (GSH-S), glutathione peroxidase (GPX), glutathione reductase (GR), glutathione S-transferase (GST), catalase (CAT), superoxide dismutase (SOD), ascorbate peroxidase (AP), tocopherol cyclase (TOCC), glycolate oxidase (GOX), ATP-binding cassette transporter (ABC). [file 12864_2015_1574_MOESM2_ESM.pdf]

DECL STAT EXP

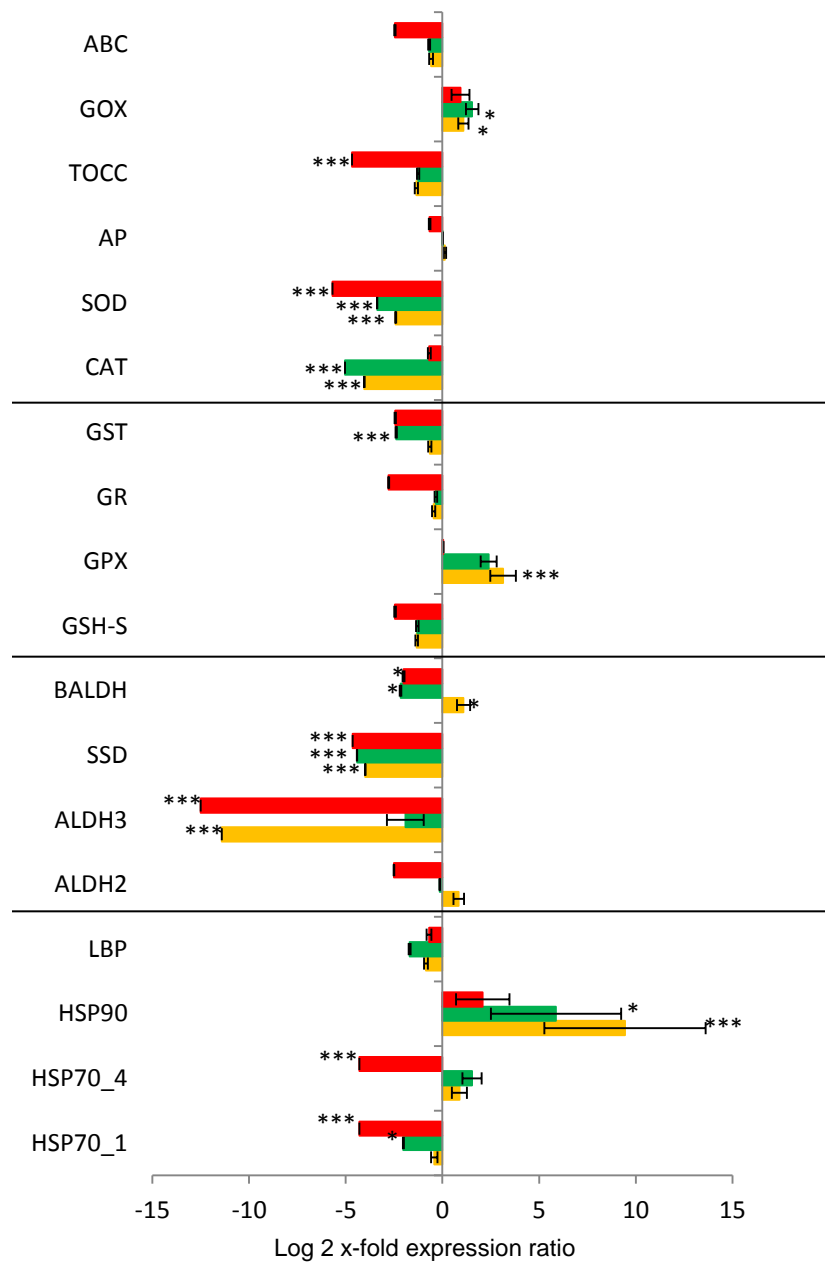

Supplement: Additional file 3: — Gene expression in Skeletonema marinoi grown in P-starved medium. Expression levels of heat shock proteins, aldehyde dehydrogenases, glutathione-related enzymes and other antioxidants in S. marinoi during exponential, stationary and declining growth phases (EXP, STAT, DECL) in P-starved medium, using S. marinoi grown in complete medium as control (x-axis; * for p < 0.05, ** for p < 0.01 and *** for p < 0.001). Data are represented as log2 x-fold expression ratio ± SD. Gene abbreviations used are: Heat shock proteins (HSP), luminal binding protein (LBP), aldehyde dehydrogenases (ALDH), succinate dehydrogenase (SSD), betaine ALDH (BALDH), glutathione synthase (GSH-S), glutathione peroxidase (GPX), glutathione reductase (GR), glutathione S-transferase (GST), catalase (CAT), superoxide dismutase (SOD), ascorbate peroxidase (AP), tocopherol cyclase (TOCC), glycolate oxidase (GOX), ATP-binding cassette transporter (ABC). [file 12864_2015_1574_MOESM3_ESM.pdf]
